# Establishment of a prognostic risk model for prostate cancer based on Gleason grading and cuprotosis related genes

**DOI:** 10.1007/s00432-024-05899-9

**Published:** 2024-08-01

**Authors:** Haicheng Wang, Meiyi Xie, Yuming Zhao, Yong Zhang

**Affiliations:** 1 Department of Urology, Hebei Medical University, Shijiazhuang, China; 2 Department of Urology, National Cancer Center/National Clinical Research Center for Cancer/Cancer Hospital, Chinese Academy of Medical Sciences and Peking Union Medical College, Beijing, China; 3 Department of Urology, Qinhuangdao First Hospital, No. 258 Wenhua Road, Haigang District, Qinhuangdao, 066000 China

**Keywords:** Prostate cancer, Cuproptosis, Gleason grading system, STX3, Nonnegative matrix factorization

## Abstract

**Introduction:**

Prostate cancer (PCa) is common in aging males, diagnosed via the Gleason grading system. The study explores the unexamined prognostic value of cuprotosis, a distinct cell death type, alongside Gleason grades in PCa.

**Methods:**

We explored Cuprotosis-related genes (CRGs) in prostate cancer (PCa), using NMF on TCGA-PRAD data for patient classification and WGCNA to link genes with Gleason scores and prognosis. A risk model was crafted via LASSO Cox regression. STX3 knockdown in PC-3 cells, analyzed for effects on cell behaviors and tumor growth in mice, highlighted its potential therapeutic impact.

**Results:**

We identified five genes crucial for a prognostic risk model, with higher risk scores indicating worse prognosis. Survival analysis and ROC curves confirmed the model’s predictive accuracy in TCGA-PRAD and GSE70769 datasets. STX3 was a key adverse prognostic factor, with its knockdown significantly reducing mRNA and protein levels, impairing PC-3 cell functions. In vivo, STX3 knockdown in PC-3 cells led to significantly smaller tumors in nude mice, underscoring its potential therapeutic value.

**Conclusion:**

Our prognostic model, using five genes linked to Gleason scores, effectively predicts prostate cancer outcomes, offering a novel treatment strategy angle.

**Supplementary Information:**

The online version contains supplementary material available at 10.1007/s00432-024-05899-9.

## Introduction

Prostate cancer (PCa) is a prevalent malignancy impacting the urinary tract. In developed Western nations, prostate cancer ranks as the second most common cause of cancer-related deaths in men (Jemal et al. 2016). Similarly, in China, there were over 60,000 reported cases of prostate cancer annually in 2015 (CHEN et al. 2016). While early-stage PCa patients often have a favorable prognosis and low mortality rates (SEIKKULA H A, KAIPIA A J, RYYNÄNEN et al. 2018), a notable subset develops resistance to androgen deprivation therapy (ADT), leading to a worsened prognosis (SAAD 2019).

Presently, prostate cancer (PCa) is acknowledged for its considerable diversity, manifesting varied clinical as well as histological features, including multifocal tumor distribution and differing Gleason Patterns (GPs) across lesions (NWOSU et al. 2001). Wang et al. employed weighted correlation network analysis (WGCNA) to uncover candidate genes associated with Gleason scoring in PCa, marking the first attempt of its kind. By constructing and assessing a least absolute shrinkage and selection operator (LASSO) Cox regression model, they explored the prognostic significance of Gleason scoring system-related genes in PCa, leveraging data from the TCGA-PRAD as well as MSKCC cohorts (WANG 2020). This research underscored the crucial role of GP as a clinical predictor in PCa prognosis. It’s important to note that GP assessments rely on visual observation, potentially leading to discrepancies among observers analyzing the same pathological Sect. (ALLSBROOK W C et al. 2001). Furthermore, due to PCa’s pronounced heterogeneity and challenges in biopsy collection, patients with identical GP may present significant variations in clinical prognosis (SHEN and ABATE-SHEN 2010). It’s evident that the Gleason scoring system alone is insufficient for contemporary needs, emphasizing the clinical imperative for more objective and precise indicators to assist in prognosticating PCa patients.

Copper (Cu) is a vital mineral nutrient essential for various biological processes, including energy metabolism, mitochondrial respiration, iron uptake, and antioxidant and detoxification mechanisms (RUIZ and ELORZA 2021). Imbalances in copper levels, whether excessive or deficient, are closely associated with the onset and advancement of several tumors (KOWALSKI 1994; LI 2020). Recent studies indicate that direct copper binding to lipid acylated components of the tricarboxylic acid (TCA) cycle can trigger toxic protein stress, resulting in cell death—a phenomenon referred to as cuproptosis (Milla Villeda et al. 1997). Cuproptosis has shown significant associations with prognosis across different cancers and the onset of various diseases, including hepatocellular carcinoma as well as bladder cancer (DIOT and GAGNADOUX 1997; WANG M Q et al. 1997). The expression levels of iron oxidation reductase protein 1 (FDX1), a critical regulator of cuproptosis, have also demonstrated significant correlations with immune microenvironment and drug resistance in diverse cancer types (Pavlov 1998). In a prostate cancer (PCa)-related study, Jin et al. investigated the prognostic implications of cuproptosis in PCa by analyzing the mutational profile of ten cuproptosis-related genes using bioinformatics algorithms. Their findings also revealed differences in gene expression, immune infiltration, immunotherapy response, and chemoresistance among distinct cuproptosis subtypes in PCa patients (SAKAI and AKIMA 1998).

This study uses WGCNA to find genes in the TCGA cohort linked to cuproptosis and Gleason scores, then creates a prognostic model for prostate cancer. It aims to improve understanding of cuproptosis and Gleason scores’ prognostic value, promoting early detection and better patient outcomes.

## Materials and methods

### Data sources

Normalized gene expression data from RNA sequencing (RNA-seq), which included read counts as well as fragments per kilobase million (FPKM) values of genes, were acquired from the TCGA Prostate Cancer (PRAD) dataset (TCGA-PRAD) (https://portal.gdc.cancer.gov/) available on the University of California Santa Cruz (UCSC) Xena database (https://xenabrowser.net/datapages/). The TCGA-PRAD dataset comprised 499 prostate cancer (PCa) samples, with 338 of them having corresponding disease-free interval (DFI) information, along with 52 normal samples, as indicated by the dataset annotation. The GSE70769 dataset, submitted by Ross-Adams H et al., (Ross-Adams et al. 2017) contains 92 prostate cancer (PCa) samples without biochemical recurrence (BRF). This dataset was obtained from the Gene Expression Omnibus (GEO) database (https://www.ncbi.nlm.nih.gov/geo/) utilizing the GEOquery software package (DAVIS and MELTZER 2007). Additionally, 13 cuproptosis-related genes (CRGs) were identified from previous literature (LAI et al. 2022).

### Identification of the differentially expressed cuproptosis-related genes (DE-CRGs)

The TCGA-PRAD dataset underwent initial analysis for differential expression to identify genes with significant differences in expression (DEGs) (adjusted p-value < 0.05) between prostate cancer (PCa) and normal samples. This analysis utilized the ‘DESeq2’ package (Version 1.34.0) (Baker et al. 2016). Notably, differentially expressed cuproptosis-related genes (DE-CRGs) were identified by intersecting cuproptosis-related genes (CRGs) with the DEGs obtained. Heatmaps and boxplots illustrating the expression patterns of DE-CRGs were generated using ‘pheatmap’ (Version 1.0.12) (MAO et al. 2022) and ‘ggpubr’ (Version 0.4.0) (ZHANG and SHEN 2020), respectively. The positional relationships of DE-CRGs within genomic intervals were visualized using the ‘RCircos’ tool (ZHANG and MELTZER 2013). Furthermore, the correlation among these DE-CRGs was graphically depicted using the ‘corrplot’ package (Version 0.92) (ZHANG et al. 2021).

### Nonnegative matrix factorization (NMF) analysis

Using gene expression data (FPKM values) of the identified DE-CRGs alongside associated disease-free interval (DFI) information, we utilized the Nonnegative Matrix Factorization (NMF) R package (Version 0.21.0) (GAUJOUX 2010) to divide the 338 prostate cancer (PCa) patients with DFI information from the TCGA-PRAD dataset into distinct clusters. Next, we compared the expression profiles of the DE-CRGs across these clusters. Additionally, survival analysis for the different clusters was carried out utilizing the survival package (Version 3.3-1) (WANG 2023).

### Identification of the cluster with poorer prognosis-related genes

We carried out Weighted Gene Co-expression Network Analysis (WGCNA) on the TCGA-PRAD dataset, which comprised 338 prostate cancer (PCa) patients with disease-free interval (DFI) information, using the WGCNA R package (Version 1.70-3) (LANGFELDER 2008). We removed outliers using hierarchical clustering and set a soft-threshold for scale-free network creation. An adjacency matrix from FPKM data was transformed into a topological overlap matrix (TOM) to cluster correlated genes. Pearson correlation identified a key module (KM1) significantly associated with our clinical trait (|Cor| > 0.3, p.adj < 0.05), and we analyzed its gene significance (GS) and module membership (MM).

### Identification of the Gleason score-related genes

To assess the prognostic value of combining cuproptosis with Gleason grades in PCa, we performed WGCNA on TCGA-PRAD data from 499 patients, aiming to identify Gleason score-related genes. After removing outliers and choosing a soft-threshold, we clustered genes into modules based on expression patterns and used Pearson correlation to link these modules with Gleason scores, identifying a key module (KM2) for further analysis.

### Identification and evaluation of prognostic risk model

We overlapped genes in KM1 and KM2 to find candidate genes, investigating their functions and pathways via GO and KEGG analyses. Using univariate and LASSO Cox regression, we identified prognostic markers from the TCGA-PRAD and GSE70769 datasets for training and validation. Patients were classified into high- and low-risk groups based on median risk scores, with risk curves visualized and populations differentiated by PCA. Survival differences between groups were analyzed using Kaplan-Meier curves. (ZHU et al. 2022). Receiver operating characteristic (ROC) curves, evaluating the discrimination accuracy of the gene-based prognostic risk model, were generated using the ‘timeROC’ package (Version 0.4) (BLANCHE and DARTIGUES J F, JACQMIN-GADDA 2013).

### Exploration of the potential mechanism associated with the prognostic risk model

Taking into account the underlying pathological mechanisms of prostate cancer (PCa), we further identified differentially expressed genes (DEGs) between high- and low-risk groups in the TCGA-PRAD dataset. Subsequently, these DEGs underwent functional enrichment analyses through the ‘org.Hs.eg.db’, ‘cluster Profiler’ (Version 4.2.2), and ‘enrichplot’ (Version 1.14.2) R packages (LI W H et al. 2021; XIONG et al. 2022).

### Statistical analysis

All analyses were conducted utilizing the R programming language (https://www.r-project.org/). The wilcox.test function was utilized to compare data across different groups. Unless stated otherwise, a significance level of *p* < 0.05 was deemed statistically significant.

### Cell culture and STX3 knockdown

The PC-3 prostate cancer cell line was cultured in RPMI1640 with 10% fetal bovine serum at 37 °C and 5% CO2, with media changed every 3 days. Cells in logarithmic phase from stable passages were used for experiments. In the knockdown group, cells were infected with shRNA-lentiviral particles targeting STX3, while the control group received particles with Scramble sequences.

### Cell stimulation and RT-PCR

A total of 5 × 10^5^ PC-3 cells were seeded into 6-well plates and allowed to adhere. Before stimulation, the cells underwent three washes with phosphate buffer solution. Serum-free RPMI1640 medium was then added to replace the culture medium prior to commencing the experiment. Subsequently, RNA extraction from the cells was performed using the Trizol method, following the instructions provided with the kit (cwbio, CW2623S), in a 25 µl reaction system.

### Immunohistochemistry (IHC)

Tissue samples were fixed, embedded, and sectioned for processing, including dewaxing, hydration, and antigen retrieval. Endogenous enzyme activity was blocked, and sections were incubated with STX3 monoclonal antibody, followed by a secondary antibody. DAB chromogen was applied before microscopic examination. Sections were counterstained, dehydrated in graded ethanol, cleared in xylene, and mounted with resin.

### Western blot

Proteins were extracted, quantified, and separated on a 12% SDS-PAGE gel, then transferred to PVDF membranes. Membranes were blocked with skim milk and incubated with GAPDH and STX3 antibodies, followed by a secondary antibody. Detection was performed using an imaging system after exposure treatment.

### Clinical data collection

This retrospective study examined a cohort of 40 gastric cancer patients treated at Hebei Medical University from January 2018 to May 2022. Each patient’s PCa diagnosis was confirmed by two pathologists with unanimous histological agreement. Clinical data collected from each patient comprised age, gender, STX3 expression, tumor differentiation, smoking and drinking history, along with other pertinent information.

### CCK-8 assay

In the CCK-8 assay, PC-3 cells were seeded in a 96-well plate overnight at a density of 2 × 10^3^ cells per well. Subsequently, 20 µL of CCK8 solution (5 mg/mL) was added to each well and incubated for 4 h. The optical density at 450 nm was measured through a microplate reader.

### Wound healing assay

Cell migration in PC-3 cells was evaluated using a wound healing assay. After cells reached 90% confluency, scratches were made and wells were rinsed thrice with PBS. Fresh serum-free medium was added, and the wound was examined at 0 and 24 h post-scratch with an Olympus X71 microscope. Distance between cells was measured using ImageJ. The assay was repeated three times for statistical accuracy.

### Colony formation assay

PC-3 cells were cultured in a 6-well plate under standard conditions for two weeks at 37 °C. After incubation, the plate was washed with cold phosphate-buffered saline to remove non-adherent cells. Then, colony cells were fixed with 4% paraformaldehyde for 15 min and stained with 0.1% crystal violet at room temperature. Imaging and quantification of the colony cells were performed using a non-optical microscope.

### Transwell assay

Cell migration was assessed using Transwell assays. PC-3 cells (4 × 10^4^) were placed in the upper chamber with 5% FBS medium, and 500 µL of medium was added to the lower chamber. After 24 h, non-invaded cells were removed, and the remaining cells were fixed with paraformaldehyde and stained with crystal violet. Invaded cells were quantified under a microscope in five random fields.

## Results

### Identification of DE-CRGs and two clusters

**Fig. 1:**
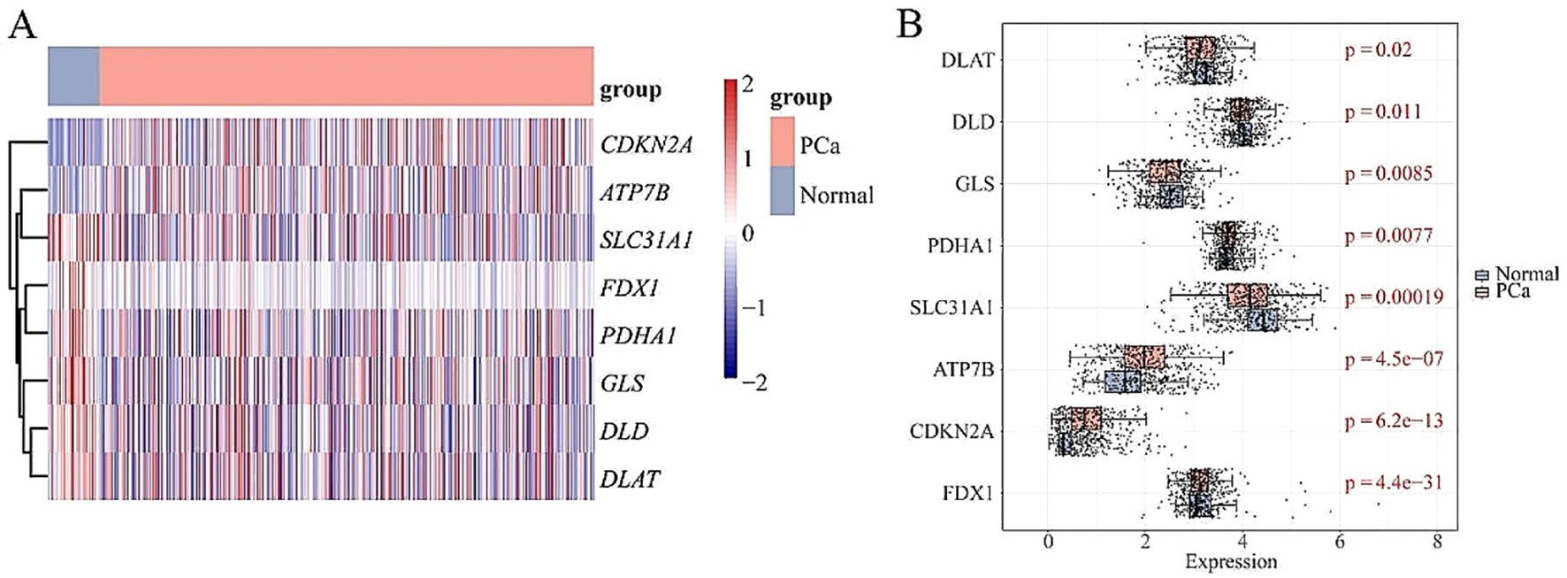
Identification of Cuprotosis-related differentially expressed genes in prostate cancer. (**A**) Heat map of Cuprotosis-related genes in prostate cancer patients. The colors in the heatmap signify the correlation, with red representing a positive correlation and blue representing a negative correlation. (**B**) Box plot of Cuprotosis-related genes in prostate cancer patients

A comprehensive analysis revealed 21,186 DEGs between PCa and normal groups, consisting of 12,229 up-regulated and 8,957 down-regulated genes. Eight DE-CRGs (DLAT, SLC31A1, PDHA1, CDKN2A, DLD, GLS, ATP7B, FDX1) were highlighted, integrating data from 13 CRGs and DEGs (Fig. 1A-B). These eight DE-CRGs are distributed across five chromosomes and exhibit notable correlations among them. Notably, DLD exhibited a significant positive correlation with DLAT, while SLC31A1 showed a marked negative correlation with PDHA1 and CDKN2A (Supplementary Fig. 1).

**Fig. 2:**
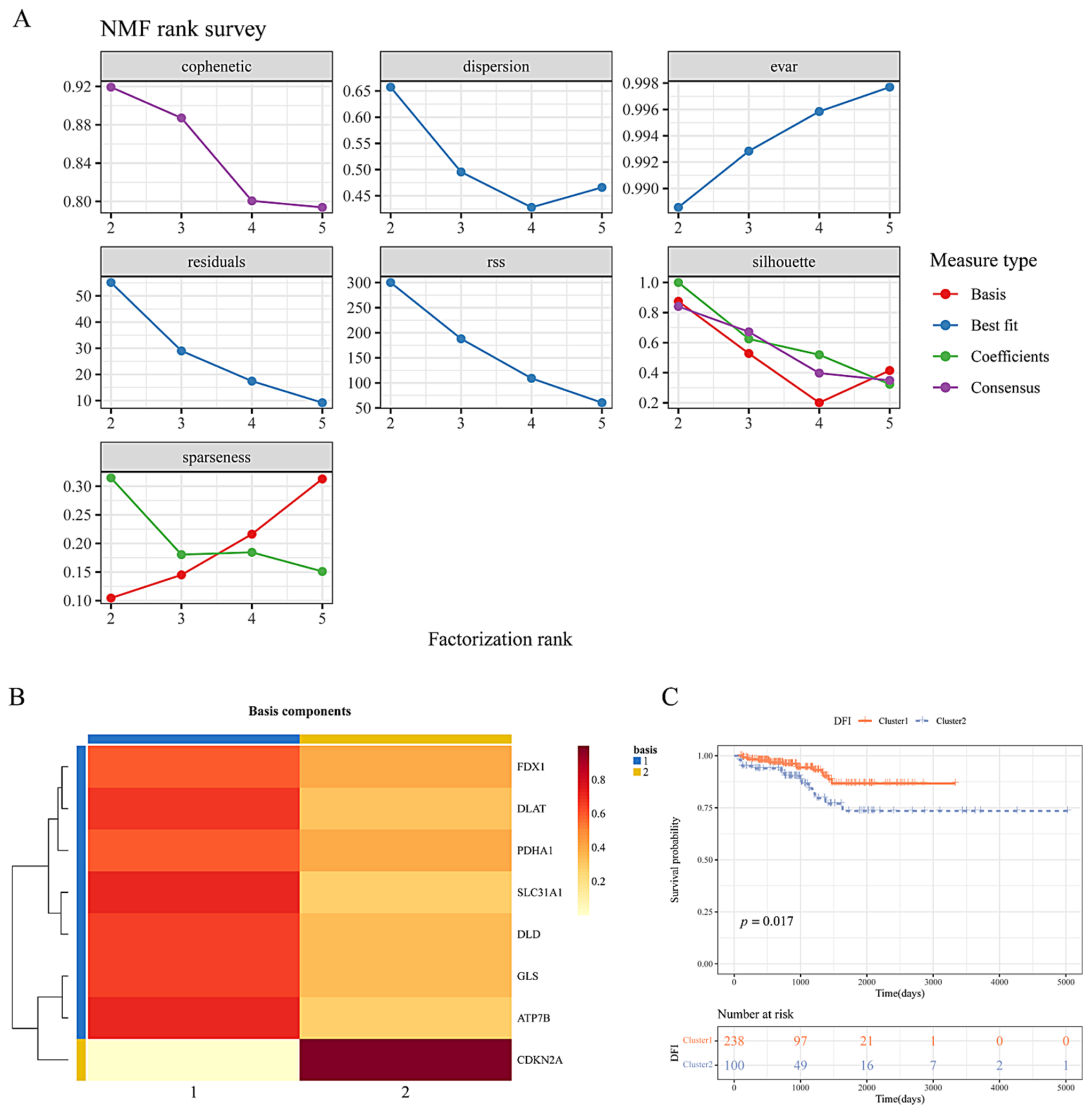
Identification of Cuprotosis clusters in prostate cancer patients. (**A**) NMF analysis results. The k value at which the resonance correlation coefficient starts to decrease was chosen and the optimal number of clusters was 2. (**B**) Expression heatmap of Cuprotosis-related genes in two types of prostate cancer patients. Blue represents the first type and yellow the second. The higher/lower the level of expression, the darker/lighter the colour. (**C**) Survival curve plots of prostate cancer patients in two clusters

Gene expressions of the mentioned 8 DE-CRGs were utilized to identify two clusters among 338 patients with survival data in the TCGA-PRAD dataset (cluster 1 = 238, cluster 2 = 100) through NMF analysis (Fig. 2A, Supplementary Table 1). Most genes exhibited consistent expression trends within the same clusters (Fig. 2B). Survival analysis revealed that patients in cluster 2 had a poorer prognosis compared to cluster 1 (Fig. 2C). Consequently, cluster 2 was chosen as the clinical trait for subsequent WGCNA analysis.

### Identification of 6561 cluster 2-related genes and 322 Gleason score-related genes

**Fig. 3:**
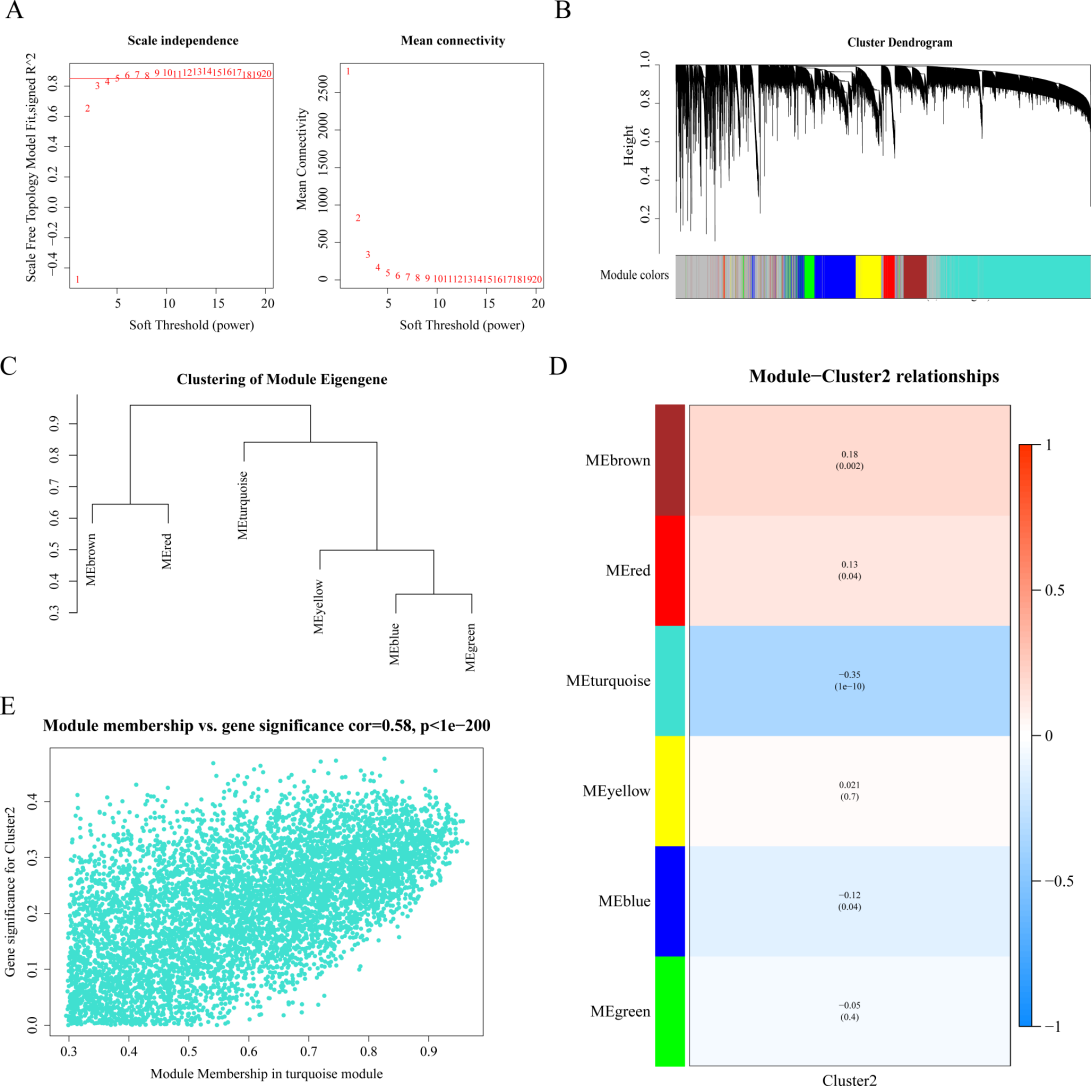
Identification of 6561 Cuprotosis clustering related genes in prostate cancer patients. (**A**) Determination of the WGCNA soft threshold. (B-C) WGCNA generates co-expression modules. (**D**) Heatmap of the correlation between 6 co-expression modules and Cluster 2. The values in the heatmap represent the correlation, and the values in the parentheses represent the p-value. Red indicates positive correlation, while blue indicates negative correlation. (**E**) Detection of the correlation between module genes and prognosis in prostate cancer patients

After excluding two outliers via sample clustering, the WGCNA analysis included 336 samples with associated DFI information from the TCGA-PRAD dataset (Supplementary Fig. 1). Selecting a soft threshold power of 6 ensured the establishment of a scale-free network (R^2 = 0.85), as shown in Fig. 3A. Subsequently, 6 co-expression modules were generated based on similar gene expression patterns (Fig. 3B-C). Among these modules, MEturquoise displayed the most significant negative correlation with cluster 2 (|Cor| = -0.35, p.adj < 0.0001) (Fig. 3D). Consequently, a total of 6561 genes within the MEturquoise module were identified as key module genes (referred to as cluster 2-related genes) for downstream analysis. The correlation between GS and MM is depicted in Fig. 3E, demonstrating the robust association between these module genes and cluster 2, indicating a poorer prognosis.

**Fig. 4:**
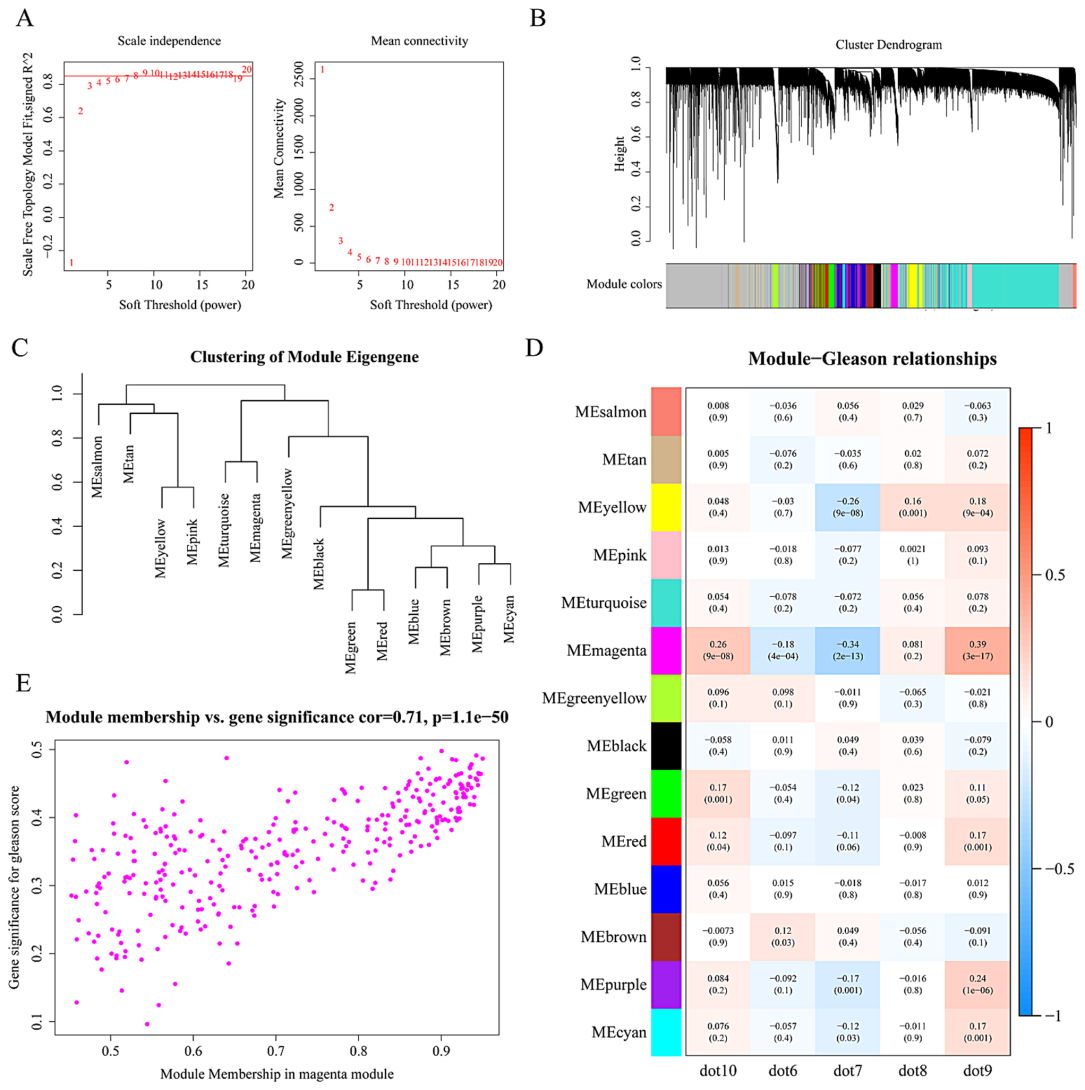
Identification of 22 Gleason score-related genes. (**A**) Determination of the WGCNA soft threshold. (B-C) Generation of co-expression modules by WGCNA. (**D**) Screening of modules related to Gleason score in prostate cancer patients. The values in the heatmap indicate the correlation, and the values in parentheses denote the p-values, with red indicating a positive correlation and blue indicating a negative correlation. (**E**) Detection of the correlation between module genes and Gleason score in prostate cancer patients

Sample clustering was conducted on 497 samples from the TCGA-PRAD dataset, excluding two outliers (Supplementary Fig. 3). A soft threshold power of 9 (Fig. 4A) was set to establish a scale-free network (R^2 = 0.85), leading to the identification of 14 co-expression modules (Fig. 4B-C). The heatmap illustrating Module-Gleason relationships revealed that MEmagenta displayed the strongest association with Gleason scores (|Cor| = 0.39, p.adj < 0.0001) (Fig. 4D). Consequently, a total of 322 genes within MEmagenta were discovered as genes related to Gleason score. The correlation between GS and MM of these genes is depicted in Fig. 4E.

### Identification and exploration of 27 candidate genes

**Fig. 5:**
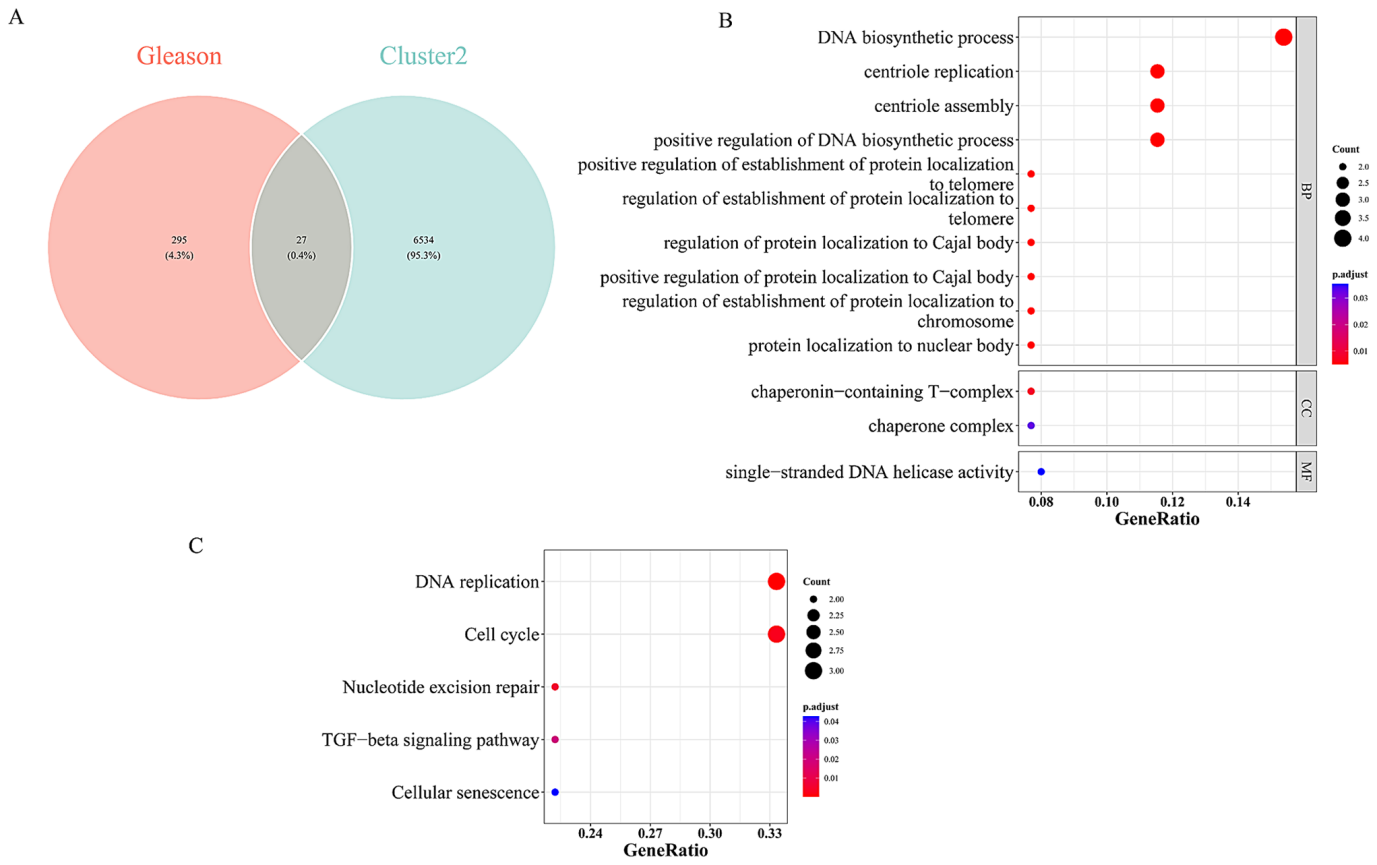
Identification and exploration of 27 candidate genes. (**A**) VENN diagram of Gleason score and genes related to copper mortality. (**B**) GO enrichment analysis of candidate genes. The x-axis shows the ratio number of genes and the y-axis shows the GO pathway terms. The size of the dots indicates the number of enriched genes in the pathway, and the colour represents the range of p-values. (**C**) KEGG enrichment analysis of candidate genes. The x-axis shows the ratio number of genes and the y-axis shows the KEGG pathway terms. The P-value of each term is colored according to the legend

Integrating Gleason score-related genes and cluster 2-related genes identified a total of 27 candidate genes (Fig. 5A, Supplementary Table 2). GO enrichment analysis revealed their enrichment primarily in various biological process (BP) terms, such as DNA biosynthetic process, centriole replication, and centriole assembly. Cellular components (CC) included chaperonin-containing T-complex and chaperone complex, while molecular functions (MF) encompassed single-stranded DNA helicase activity, suggesting potential associations with these genes (Fig. 5B, Supplementary Table 3). In terms of potential KEGG signaling pathways, five terms were implicated, including DNA replication and Cell cycle (Fig. 5C, Supplementary Table 4).

### Construction and validation of the prognostic risk model

**Fig. 6:**
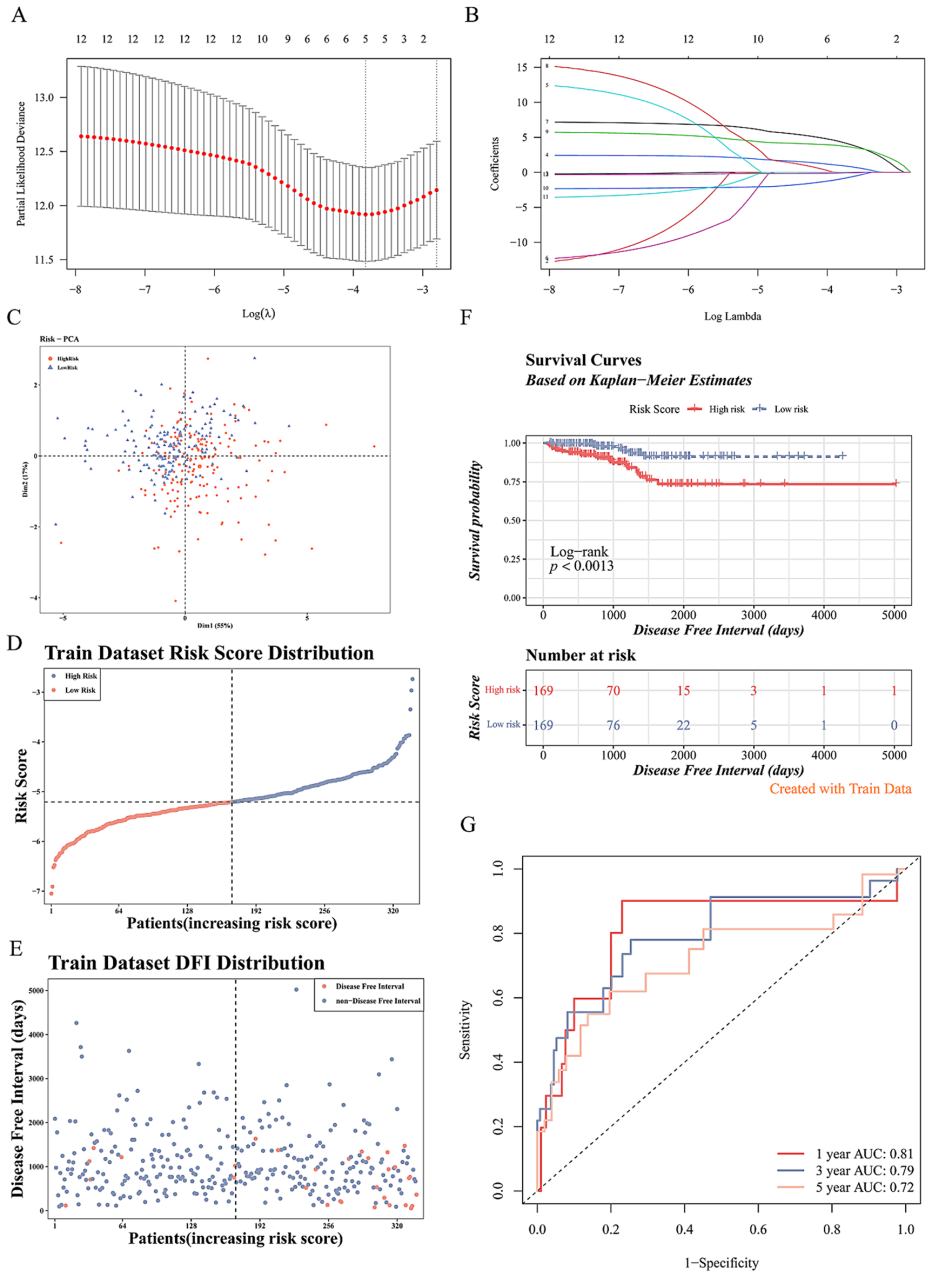
Construction of prognostic risk model. (A-B) Univariate Cox and LASSO Cox regression analysis to construct the prognostic risk model. (**C**) PCA cluster analysis. (D-E) Risk curves of the training set. (**F**) K-M survival curve analysis of patients in the training set. (**G**) ROC curve for predicting 1–5 year survival of patients in the training set

To investigate prognostic implications in PCa, we selected five out of the 27 candidate genes (STX3, CABLES2, E2F5, RALA, POLE3) utilizing univariate Cox and LASSO Cox regression analyses (*p* < 0.05) to build a prognostic risk model (Fig. 6A-B, Supplementary Table 5). Risk scores were computed for 338 patients in the training set based on the gene expression of these five prognostic genes, leading to patient stratification into high- and low-risk groups. Principal component analysis (PCA) plot revealed distinct discrimination between the two risk populations (Fig. 6C), consistent with the risk curves (Fig. 6D-E). Additionally, a significant correlation was observed between shorter DFI and higher risk scores according to Kaplan-Meier analysis (*p* < 0.0013) (Fig. 6F). Time-dependent ROC curves demonstrated that the area under the curve (AUC) for DFI at 1-, 3-, and 5-years were 0.81, 0.79, and 0.72, respectively (Fig. 6G).

**Fig. 7:**
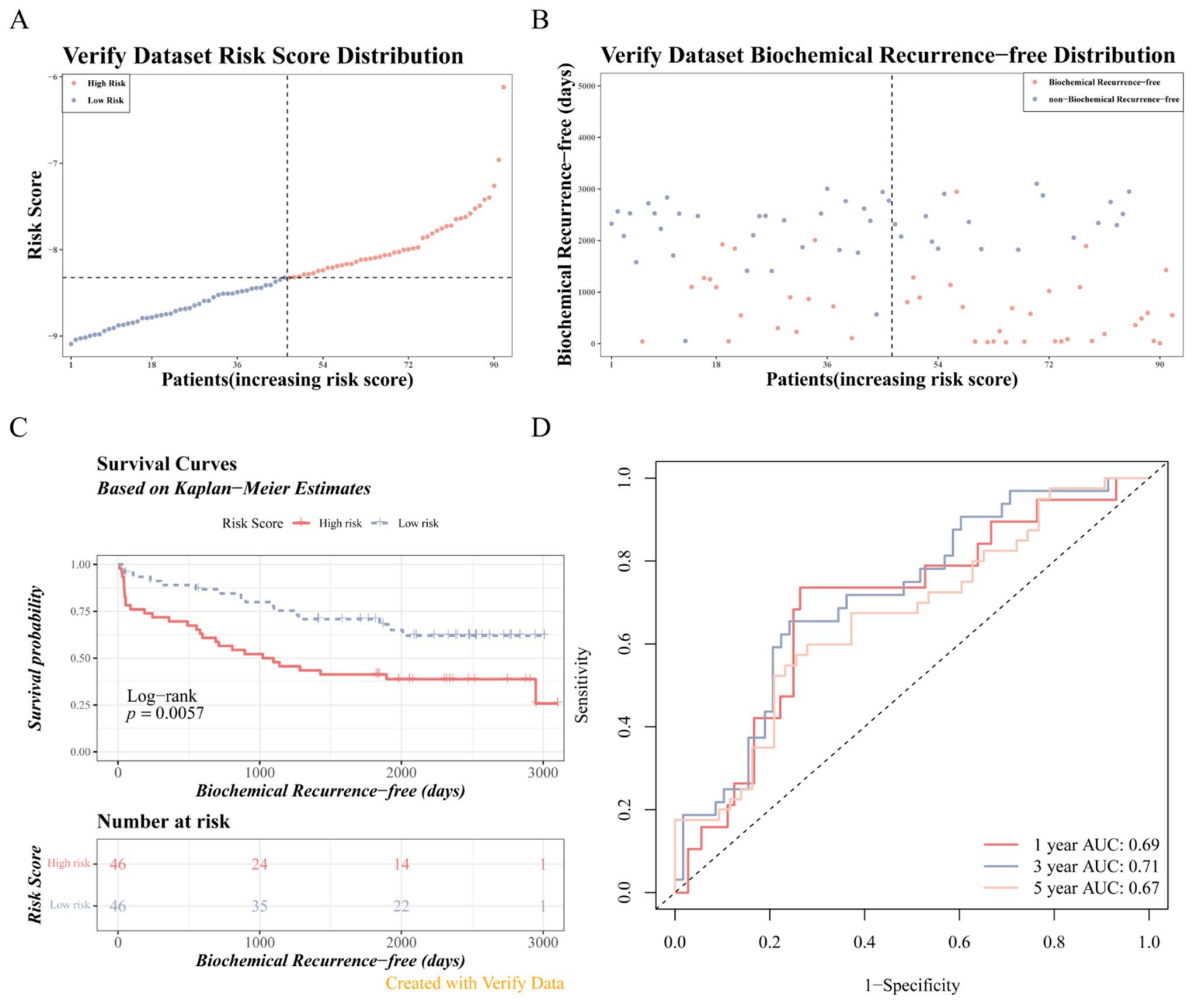
Validation of the prognostic risk model.(A-B) Risk curves in the validation set. (**C**) K-M survival curve analysis in the validation set patients. (**D**) ROC curve for predicting 1–5 year survival in the validation set patients

Likewise, we computed the risk score for each patient in the validation set (GSE70769) (Fig. 7A-B). When correlated with BRF data, it became apparent that patients in the high-risk groups demonstrated a worse prognosis (*p* = 0.0057) (Fig. 7C), with AUC values surpassing 0.6, confirming the predictive accuracy of the prognostic risk model (Fig. 7D).

### The prognostic risk model was associated with the muscle system process and cardiac muscle contraction pathway

**Fig. 8:**
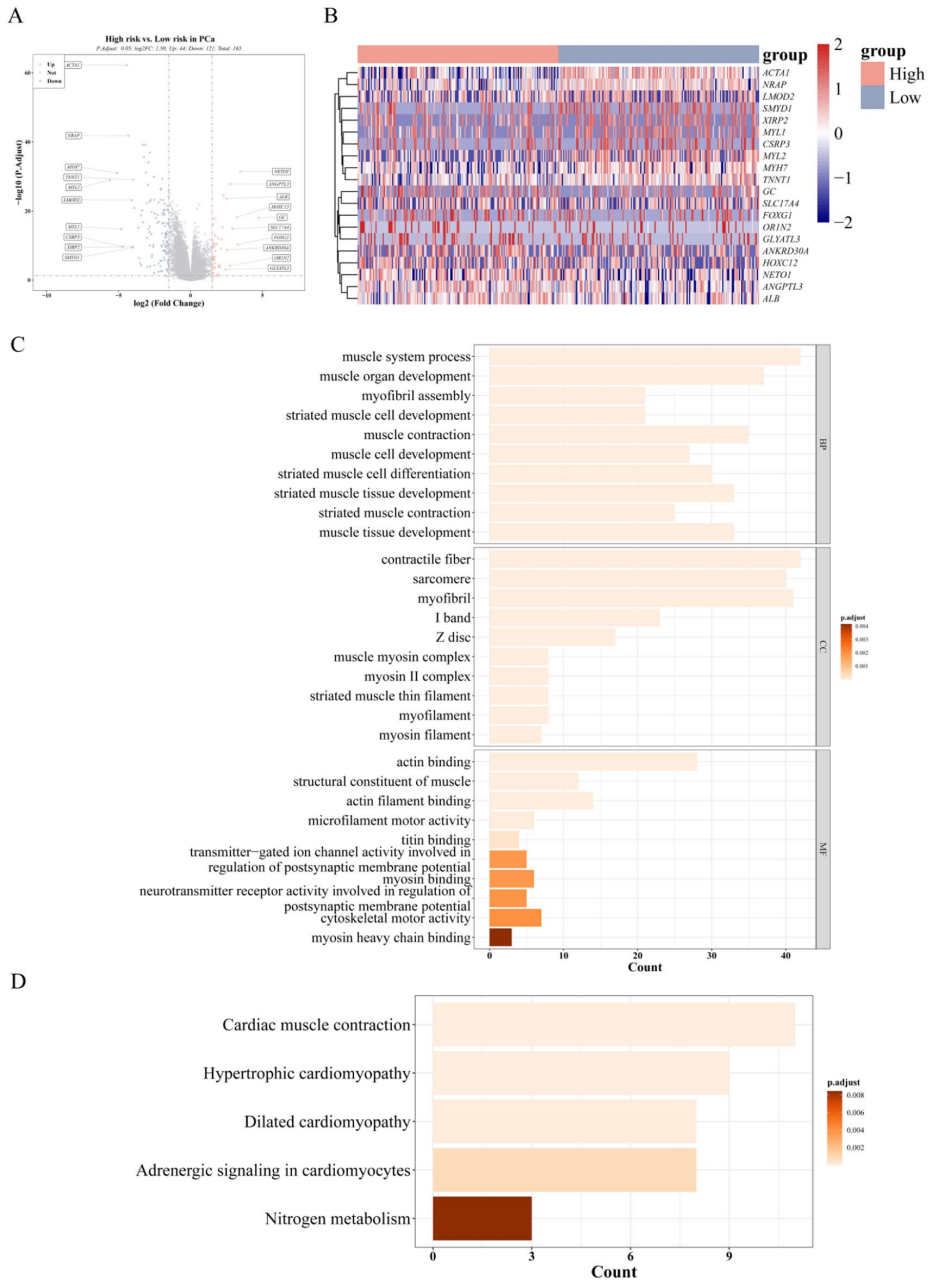
Prognostic risk model related to muscle system processes and myocardial contraction pathway. (**A**) Volcano plot of differential genes between high-risk group and low-risk group. (**B**) Heatmap of differential genes between high-risk group and low-risk group. The colors in the heatmap signify the correlation, with red representing a positive correlation and blue representing a negative correlation. (**C**) GO enrichment analyses were performed on 165 DEGs, listing the top 30 GO entries. (**D**) KEGG enrichment analysis of 165 DEGs

We investigated the underlying mechanism associated with the prognostic risk model. A total of 165 DEGs were identified between the high- and low-risk groups (|Log2FC| > 1.5, p.adj < 0.05) (Fig. 8A-B), consisting of 44 up-regulated genes and 121 down-regulated genes. Enrichment analysis revealed that the GO processes primarily involved activities related to the muscle system process, contractile fiber, and sarcomere (Fig. 8C, Supplementary Table 6). Conversely, only 5 KEGG pathways were implicated, including cardiac muscle contraction and hypertrophic cardiomyopathy (Fig. 8D, Supplementary Table 7).

### Patients with high expression of STX3 have larger tumor volumes later staging higher Gleason scores and worse prognosis

**Fig. 9:**
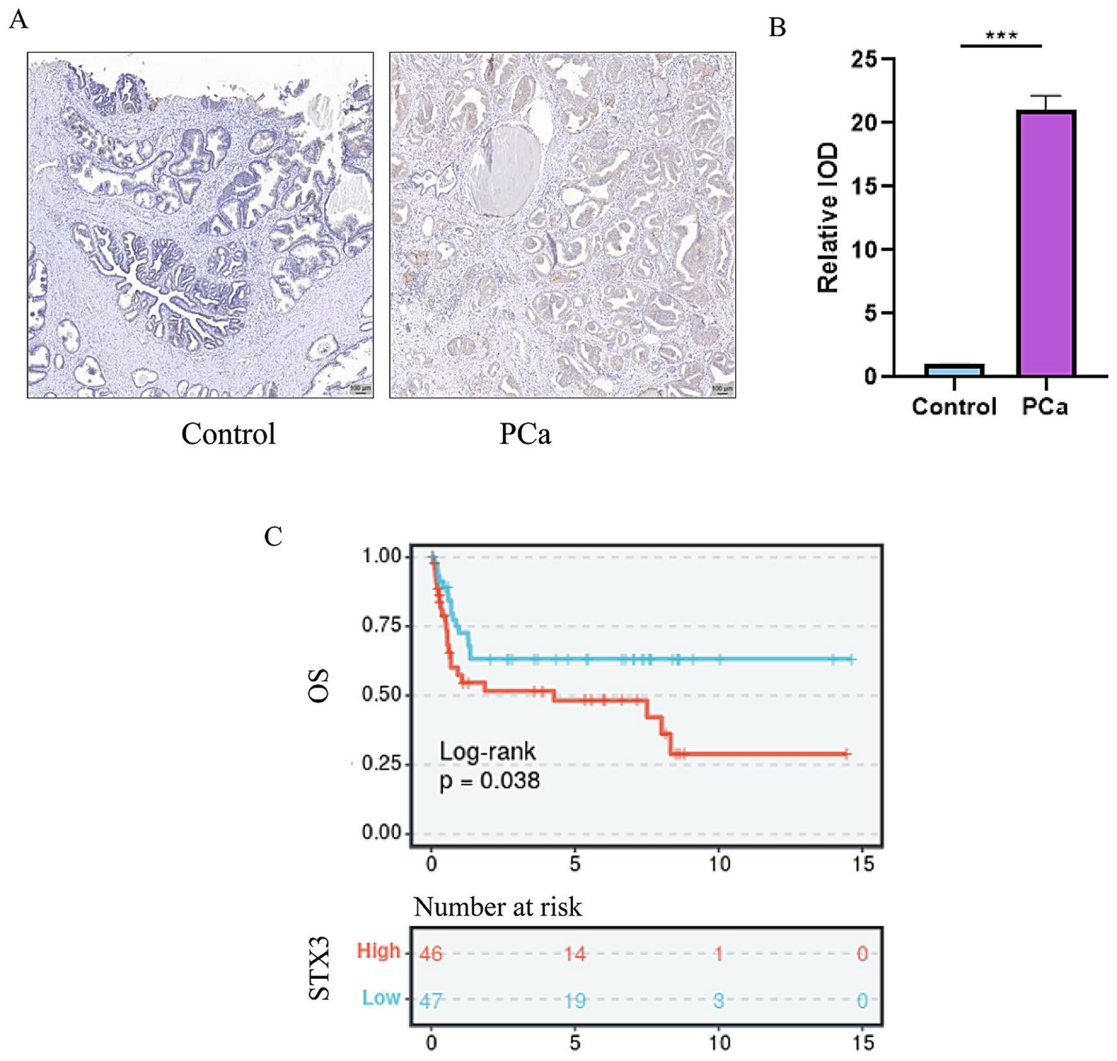
Differential expression of STX3 in PCa patients. (**A**) Immunohistochemical staining of prostate cancer group and normal control group patients. (**B**) Statistical analysis of immunohistochemical staining in prostate cancer group and normal control group patients. (**C**) Survival curve of PCa patients with different STX3 expression levels

In the research, we identified STX3 as a significant predictor for the prognosis of PCa patients, an aspect underexplored in PCa research. We collected specimens from clinical PCa patients, with their basic clinical information summarized in Table 1. Immunohistochemical staining was performed on tissues obtained from all patients (Fig. 9A). The results revealed significantly higher expression levels of STX3 in PCa patients in comparison with healthy individuals, with statistical significance (Fig. 9B). According to STX3 expression levels, patients were classified into high expression (*n* = 46) and low expression (*n* = 47) groups. Interestingly, STX3 expression was significantly elevated in PCa patients with low differentiation, stage III + IV, and lymph node metastasis compared to those with high differentiation, stage I + II, and absence of lymph node metastasis (Table 1). Additionally, patients with high STX3 expression exhibited larger tumor volumes and higher Gleason scores (Table 1). However, STX3 expression did not significantly correlate with other clinical pathological features (Table 1). The 5-year survival rates of patients with high and low STX3 expression were 49.29% and 68.17%, respectively. Moreover, the total survival period of patients with low STX3 expression was notably higher than that of patients with high STX3 expression (Fig. 9C).

**Table 1:** Comparison of STX3 expression levels in PCa patients with different pathological features(n(%))

Clinical Features	*n*	STX3		χ2		*P*
		LOW(*n* = 47)	HIGH(*n* = 46)			
Smoking history						
Yes	57	30(52.63%)	27(47.37%)	0.097	0.724	
No	36	17(47.22%)	19(52.78%)			
AGE						
< 60	47	26(55.32%)	21(44.68%)	0.587	0.422	
≥ 60	46	21(45.65%)	25(54.35%)			
Malignant medical history						
Yes	37	17(45.95%)	20(54.05%)	0.137	0.711	
No	56	30(53.57%)	26(46.43%)			
Residual tumor						
R0	33	16(48.48%)	17(51.52%)			
Rx/R1/R2	35	19(54.29%)	16(45.71%)	2.627	0.123	
NA	25	12(48%)	13(52%)			
Tumor diameter						
≤ 3 cm	51	32(76.19%)	10(23.81%)	14.271	< 0.001	
> 3 cm	42	15(29.41%)	36(70.59%)			
TNM						
I+II	43	37(74%)	13(26%)	17.579	< 0.001	
III+III	50	10(23.26%)	33(76.74%)			
Infiltration Depth						
Superficial	43	20(46.51%)	23(53.49%)	0.473	0.423	
Deep	50	27(54%)	23(46%)			
Lymph Node Metastasis						
Yes	55	37(67.27%)	18(32.73%)	10.072	< 0.001	
No	38	10(26.32%)	28(73.68%)			
Degree of Differentiation						
Low Differentiation	51	16(31.37%)	35(68.63%)	12.574	< 0.001	
Intermediate/High Differentiation	42	31(73.81%)	11(26.19%)			
Gleason Score						
6	26	20(76.92%)	6(23.08%)	13.572	< 0.001	
7	21	15(71.43%)	6(28.57%)			
8	16	5(31.25%)	11(68.75%)			
9–10	30	7(23.33%)	23(76.67%)			

### STX3 promotes the proliferation, migration, and invasion of prostate cancer cells

**Fig. 10:**
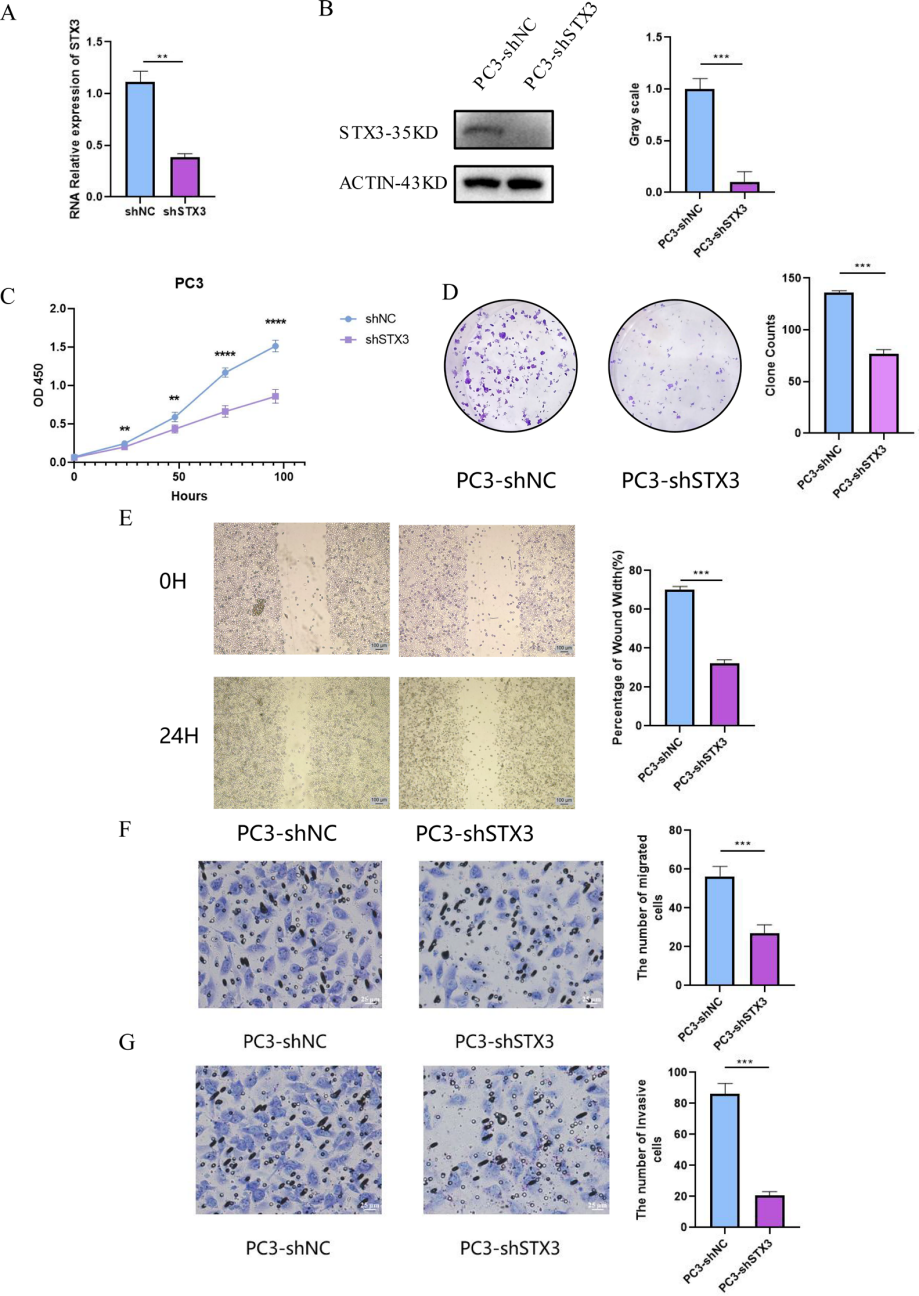
STX3 inhibits proliferation migration and invasion of prostate cancer cells in vitro. (**A**) Relative expression levels of STX3 mRNA in the sh-STX3 group and sh-NC group. (**B**) Protein expression levels of STX3 in the sh-STX3 group and sh-NC group. (**C**) OD450 values of CCK8 experiments in sh-STX3 group and sh-NC group. (**D**) Colony formation results in sh-STX3 group and sh-NC group. (**E**) Wound healing rate at 24 h in the sh-STX3 group and sh-NC group. (**F**) Number of migrated cells in the sh-STX3 group and sh-NC group. (**G**) The number of invading cells in the sh-STX3 group and sh-NC group

To explore STX3’s influence on prostate cancer cell behaviors like proliferation, migration, and invasion, we established PCa cell lines with STX3 knockdown. In the PC-3 cell line, STX3 mRNA expression notably decreased (independent samples t-test, *P* < 0.01), as shown in Fig. 10A. Western blot analysis further confirmed a significant reduction in STX3 expression in the sh-STX3 group compared to the sh-NC group (Fig. 10B), validating the efficacy of STX3 knockdown.

CCK-8 assays conducted 24 h post-transfection exhibited a notably reduced proliferation rate within the sh-STX3 group in comparison with the sh-NC group in the PC-3 cell line (Fig. 10C). Similarly, clonogenic assays demonstrated a significant decrease in clonogenic potential within the sh-STX3 group in comparison with the sh-NC group (Fig. 10D). These findings collectively suggest that downregulation of STX3 suppresses the proliferation of PCa cells.

Migration assays (Fig. 10E-F) demonstrated a significant reduction in the migration rate of the sh-STX3 group compared to the sh-NC group in the PC-3 cell line. This suggests that downregulating STX3 expression suppresses the migration ability of PCa cells. Additionally, Transwell invasion assays conducted in the PC-3 cell line revealed a significantly decreased number of cells passing through the Transwell chamber in the sh-STX3 group compared to the sh-NC group (Fig. 10G), indicating that STX3 downregulation attenuates the invasion capability of PCa cells.

### STX3 promotes the proliferation of prostate cancer cells in mice

**Fig. 11:**
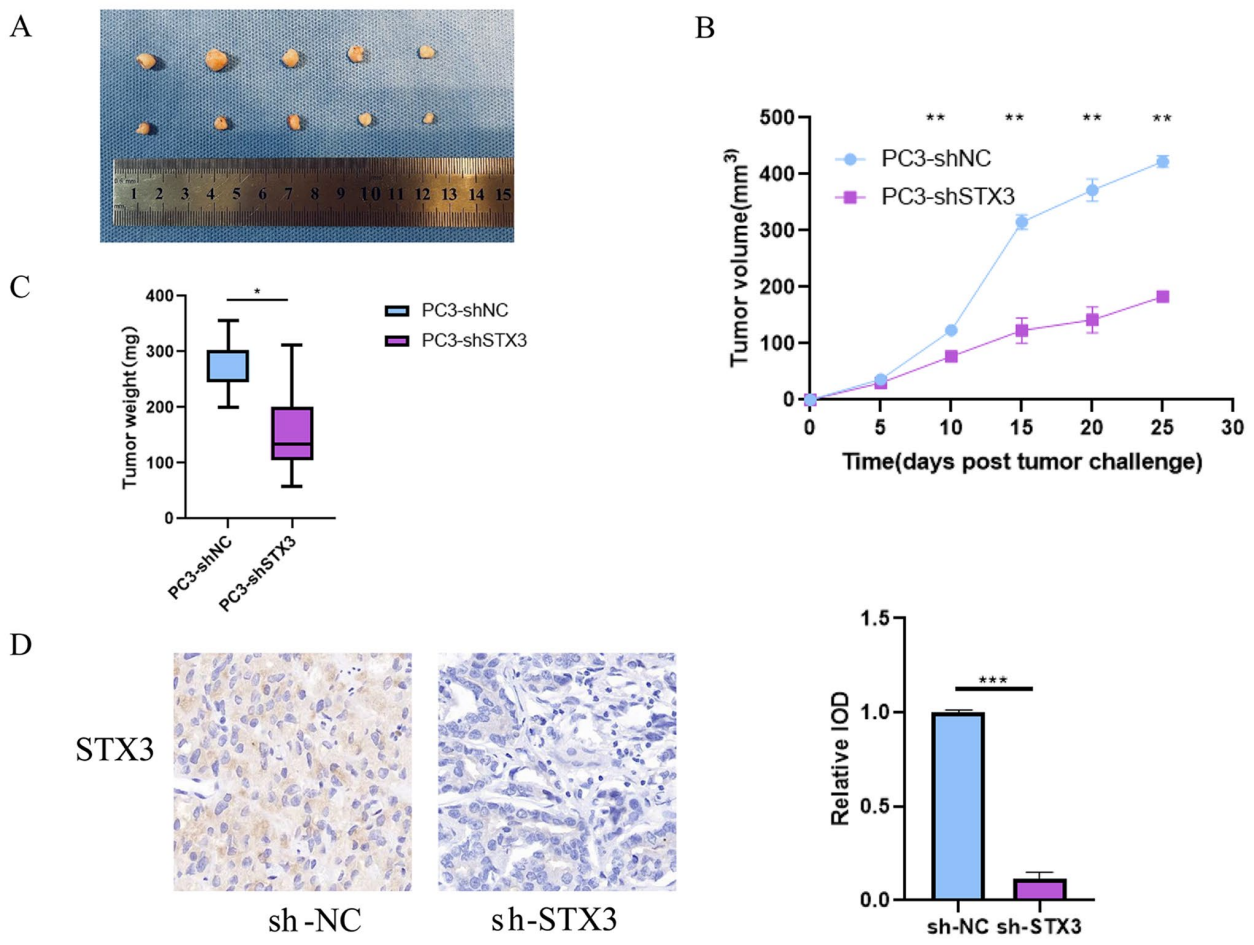
Suppression of STX3 Expression Reduces Prostate Cancer Cell Growth and Tumor Burden in Nude Mouse Models. (**A**) Tumor size in sh-STX3 group and sh-NC group mice. (**B**) Tumor volume size in sh-STX3 group and sh-NC group mice. (**C**) Difference in tumor weight between sh-STX3 group and sh-NC group mice. (**D**) Immunohistochemical staining of STX3 mouse tumor and its statistical results

Using the PC-3 cell line, we established subcutaneous tumor models in nude mice. Upon dissection, it was evident that the volume of subcutaneous tumors in the sh-STX3 group was significantly smaller than that in the sh-NC group (Fig. 11A). Quantitative analysis of tumor volume from the 10th day post subcutaneous tumor cell implantation showed a significant reduction in tumor volume in the sh-STX3 group compared to the sh-NC group (Fig. 11B). Furthermore, the weight of each tumor tissue post-dissection revealed a substantially lighter tumor weight in the sh-STX3 group compared to the sh-NC group (Fig. 11C). Immunohistochemical staining confirmed STX3 expression in tumor tissues (Fig. 11D).

In conclusion, suppressing STX3 expression led to diminished PCa cell growth in nude mice, resulting in decreased tumor volume and burden on the animals.

## Discussion

Globally, prostate cancer (PCa) stands as the most prevalent non-skin cancer among men, with an annual incidence of 1.6 million cases and leading to 366,000 deaths (TORRE L A, BRAY et al. 2015). Despite significant progress in PCa treatment, it continues to present a considerable challenge for male patients (WANG and ZHAO 2018).

In recent years, advancements in bioinformatics have enabled the discovery of promising tumor markers for diagnosing and predicting prostate cancer (PCa) outcomes. Geng X et al. utilized LASSO regression on PCa datasets from TCGA-PRAD, MSKCC, and various GEO databases, identifying five matrix metalloproteinases (MMPs)-related genes strongly associated with PCa prognosis. Their study suggests that MMPs may impact PCa prognosis by influencing pathways such as Pid integrin1, the G2M checkpoint, and response to growth factors (GENG et al. 2020).

In another study, Li J et al. identified 66 differentially expressed autophagy-related lncRNAs and constructed competing endogenous RNA networks. They validated seven lncRNAs’ expression differences across TCGA-PRAD, GSE21034, and GSE94767 datasets, establishing a risk score model for PCa prognostic prediction. Their research highlights the significant role of lncRNAs in regulating autophagy in PCa (LI et al. 2021).

Similarly, the significant role of FDX1 in PCa development and immune response has been confirmed through analyses of TCGA and GTEx databases (YANG et al. 2022). Additionally, an independent study showcased that Alternol interacts with various Krebs cycle enzymes, decreasing mitochondrial respiration and ATP production in prostate cancer cells. This inhibits their proliferation, offering a novel treatment avenue for PCa (LI et al. 2019).

Acknowledging the prognostic importance of cuproptosis in various cancers as well as the predictive value of the Gleason score in PCa (WANG 2020; SAKAI and AKIMA 1998), we conducted a comprehensive analysis of two PCa-related datasets (TCGA-PRAD, GSE70769) to integrate cuproptosis with the Gleason scoring system. This innovative approach aimed to explore their combined prognostic impact and potential mechanisms in PCa.

Initially, we identified eight differentially expressed cuproptosis-related genes (DE-CRGs) by examining the overlap between differentially expressed genes (DEGs) and cuproptosis-related genes (CRGs) in prostate cancer (PCa) and normal groups. These genes are DLAT, SLC31A1, PDHA1, CDKN2A, DLD, GLS, ATP7B, and FDX1. FDX1’s function has been previously clarified, and CDKN2A’s abnormal expression has been linked to negative impacts on PCa’s disease-free survival (CAO et al. 2018). PDHA1 overexpression has been associated with PCa development through the regulation of lipid biosynthetic pathways (CHEN et al. 2018), while PDHA1 knockdown has been found to decrease survival in PCa patients by inducing overexpression of GLS1 and GLUD1. Consistent with these results, our study observed reduced PDHA1 expression in PCa patients (LI et al. 2016).

Research indicates that the dysregulation of the E2F5/p38/SMAD3 axis is associated with uncontrolled cell proliferation in prostate cancer (PCa). Specifically, the expression of the E2F5 gene was found to be elevated in PCa biopsies compared to those with benign hyperplasia, consistent with our findings. These findings also imply the therapeutic potential of artemisinin in PCa, possibly by correcting the abnormal expression of E2F5 (KARMAKAR et al. 2020).

Syntaxin 3 (STX3), a protein encoded by the human STX3 gene, has garnered attention in breast cancer research due to its significant upregulation in both gene expression and protein levels within human breast cancer tissues in comparison with adjacent non-cancerous tissues. Initial studies suggested that STX3 overexpression might inhibit breast cancer cell proliferation (NAN et al. 2018). However, this study marks the first exploration of STX3’s involvement in prostate cancer (PCa), emphasizing the need for additional validation.

RALA belongs to the RAS superfamily, serving as a highly homologous small G-protein. These proteins act as molecular switches, regulating vital cellular functions by transitioning between inactive GTP-binding and active GTP-binding states. Depending on the cancer type, RALAs may demonstrate redundant, unique, or antagonistic functions (RICHARDSON D S et al. 2022). Notably, in the PC-3 cell model, Wang et al. demonstrated that decreased RALA activity impedes the proliferation, migration, and invasion of PC3 cells, which aligns with our findings and hints at RALA potentially serving as a poor prognostic factor in prostate cancer (PCa) (WANG et al. 2015).

POLE3 is a constituent of the POLE holoenzyme, which regulates T and B cell development at various stages (SIAMISHI et al. 2020). Previous studies have recognized POLE3, among 10 other genes, as hub genes for cervical cancer, with increased expression of POLE3 associated with the advancement of cervical cancer (TU et al. 2021). However, investigations into the connection between POLE3 and the progression of prostate cancer (PCa) are scarce and warrant further exploration.

## Conclusion

Our study introduces a five-gene model integrating cuproptosis and Gleason scores to predict PCa outcomes, highlighting the need for further validation and research.

## Electronic supplementary material

Below is the link to the electronic supplementary material.

Supplementary Material 1

Supplementary Material 2

Supplementary Material 3

Supplementary Material 4

Supplementary Material 5

Supplementary Material 6

Supplementary Material 7

Supplementary Material 8

Supplementary Material 9

Supplementary Material 10

## Data Availability

All data are available in the main Excel or the supplementary materials. Transcriptomic and clinical data for all patients were obtained from TCGA Prostate Cancer (PRAD) dataset (TCGA-PRAD) (https://portal.gdc.cancer.gov/) available on the University of California Santa Cruz (UCSC) Xena database (https://xenabrowser.net/datapages/).
