# Supplementary figures and images for: Establishment of a prognostic risk model for prostate cancer based on Gleason grading and cuprotosis related genes
Source: J Cancer Res Clin Oncol. 2024 Aug 1;150(8):376. doi: 10.1007/s00432-024-05899-9 (PMC11291559; doi:10.1007/s00432-024-05899-9)

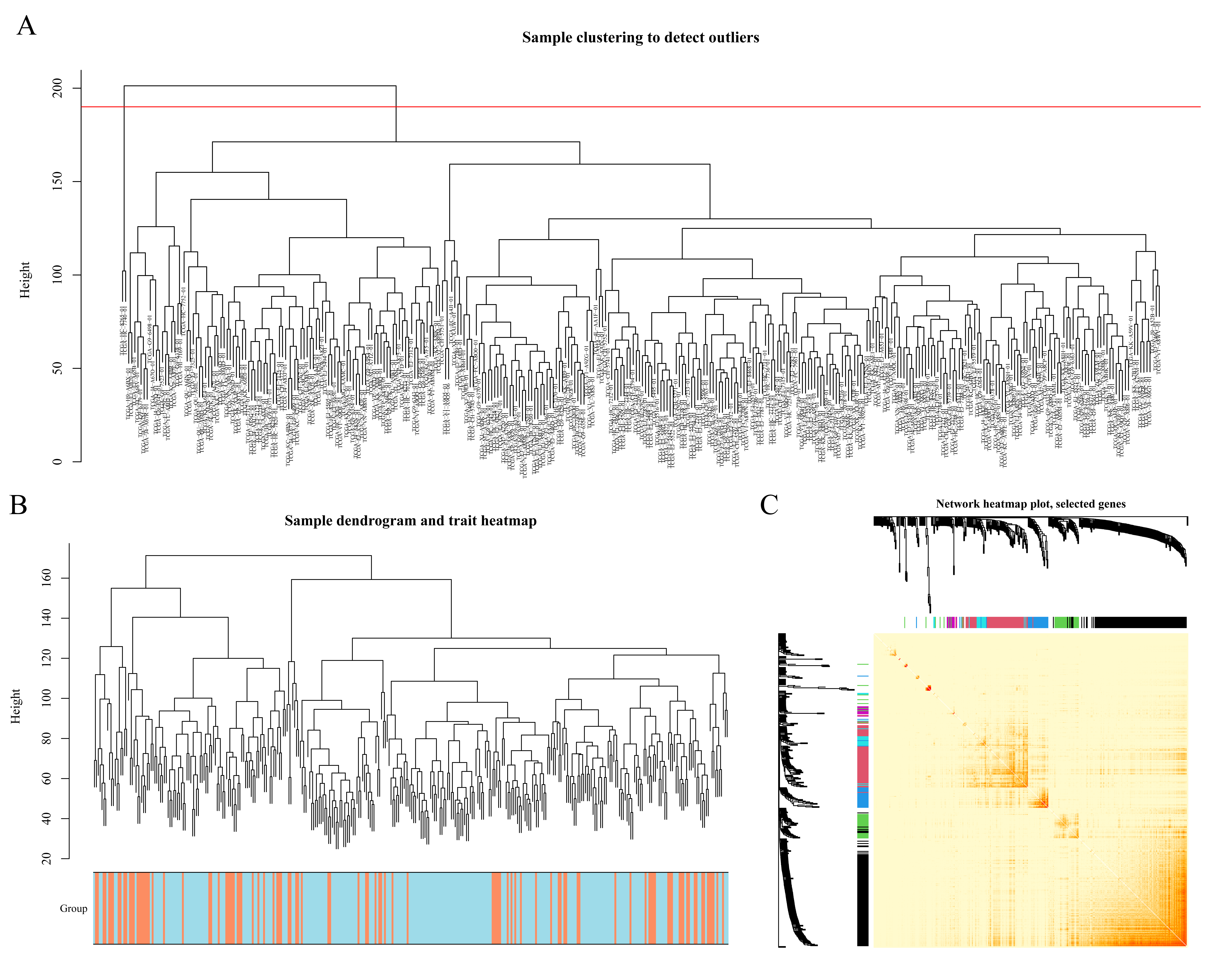

Supplement: Supplementary file 9 — Supplementary Material 9 [file 432_2024_5899_MOESM9_ESM.tif]

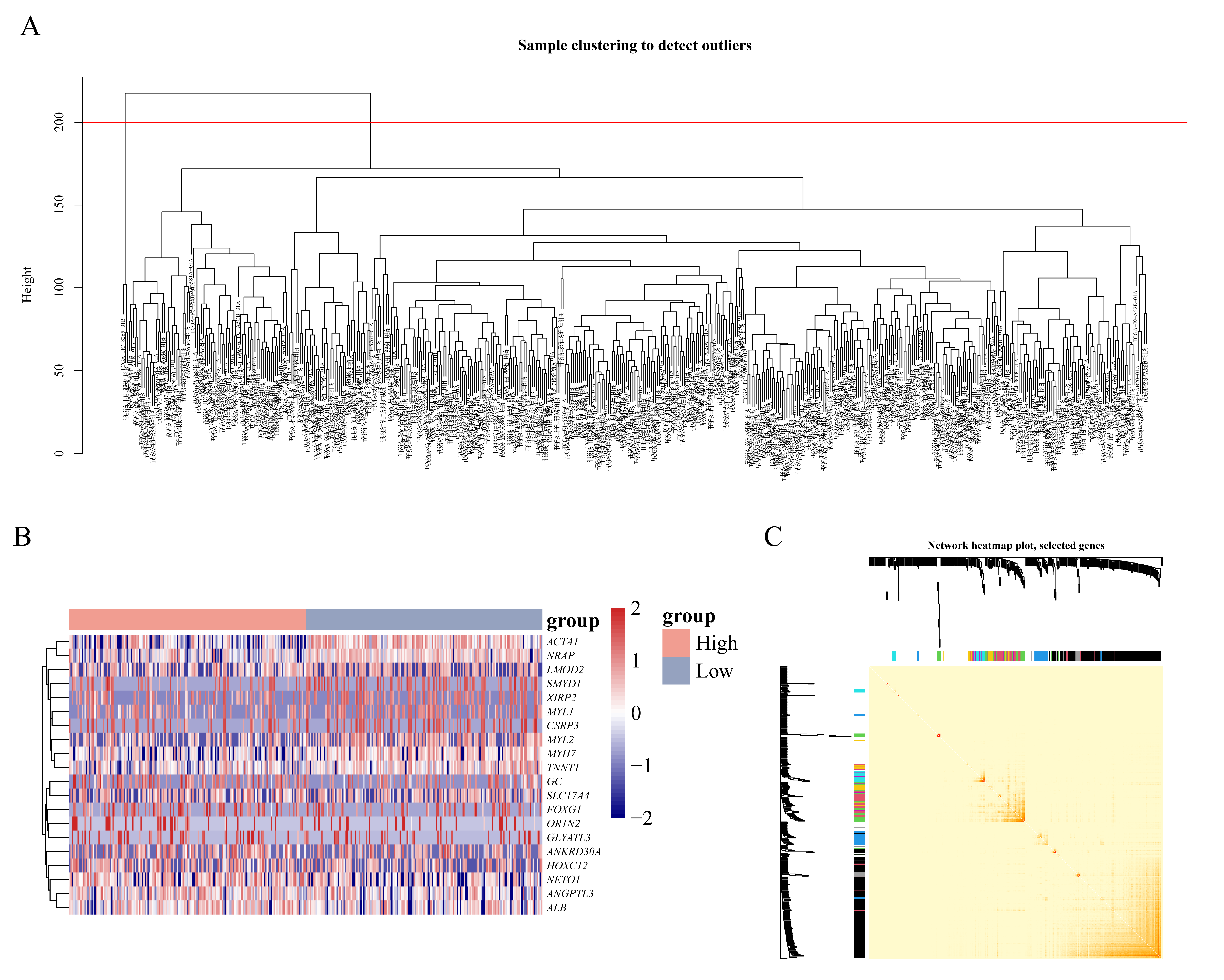

Supplement: Supplementary file 10 — Supplementary Material 10 [file 432_2024_5899_MOESM10_ESM.tif]
